# Evidence of chikungunya virus infection among febrile patients seeking healthcare in selected districts of Tanzania

**DOI:** 10.1080/20008686.2018.1553460

**Published:** 2018-12-17

**Authors:** Edson Kinimi, Mariana J. Shayo, Bisimwa N. Patrick, Samuel O. Angwenyi, Christopher J. Kasanga, Jacqueline Weyer, Petrus Jansen van Vuren, Janusz T. Paweska, Leonard E.G. Mboera, Gerald Misinzo

**Affiliations:** a Department of Veterinary Microbiology, Parasitology and Biotechnology, College of Veterinary Medicine and Biomedical Sciences, Sokoine University of Agriculture, Morogoro, Tanzania; b Center for Emerging, Zoonotic and Parasitic Diseases, National Institute for Communicable Diseases of the National Health Laboratory Services, Sandringham, Republic of South Africa; c Southern African Centre for Infectious Disease Surveillance (SACIDS) - Africa Centre of Excellence for Infectious Disease of Human and Animals, Sokoine University of Agriculture, Morogoro, Tanzania

**Keywords:** Chikungunya, seroprevalence, febrile illness, mosquito-borne, Tanzania

## Abstract

**Introduction**: Chikungunya virus (CHIKV) infection is an emerging mosquito-borne disease that has been associated with frequent epidemics in the world. However, there is a dearth of information on its magnitude and associated risk factors in Tanzania.

**Objective**: A study was conducted to determine seroprevalence of CHIKV among febrile patients seeking medical care at health facilities in Karagwe, Sengerema, Kilombero and Kyela districts.

**Methods**: Structured questionnaires were administered and 728 serum samples were collected between May and June, 2015 and tested for the presence of CHIKV-IgM and IgG-specific antibodies using Enzyme-linked immunosorbent assay.

**Results and discussion**: The common clinical characteristics exhibited by outpatients were fever, headache and joint pains (100%, 70%, and 68.3% respectively). Out of 728 outpatients screened for CHIKV, 105 (14%) tested CHIKV IgG positive whilst 11 (1.5%) tested CHIKV IgM positive. Chikungunya seropositivity was significantly higher than previously reported in Tanzania. The most affected age group was 20–29 years. Our results indicate that CHIKV infection is prevalent and contributes to the burden of febrile illnesses in Tanzania. The seroprevalence varies between districts, reflecting variation in mosquito vector transmission dynamics in different parts of the country.

**Abbreviations:** CHIKV: Chikungunya virus; EDTA: Ethylenediaminetetraacetic acid; ELISA: Enzyme-linked immunosorbent assay; IgG: Immunoglobulin G; IgM: Immunoglobulin M; NIMR: National Institute for Medical Research; RU: Relative Units; SACIDS: Southern African Centre for Infectious Disease Surveillance; USA: United States of America

## Background

Chikungunya is a mosquito-borne virus infection transmitted to humans through the bite of infected mosquitoes of the genus *Aedes* [1]. The Chikungunya virus (CHIKV) was first recognized and isolated in 1952 from Makonde Plateau in southern Tanzania [2,3]. The word ‘chikungunya’ originates from Makonde language referring to the bending stance in afflicted patients due to musculoskeletal pain [1]. Chikungunya is an illness characterized by fever, severe arthralgia, rash, headache, malaise, muscle aches and retro-orbital pain [4,5]. Although the disease is rarely fatal, it is associated with significant morbidity in which arthralgia can be debilitating and prolonged [1,6,7]. Mother-to-child transmission of CHIKV resulting in neurologic and haemorrhagic complications in affected infants has been reported [8].

Chikungunya is currently of a global public health concern due to its continued spread and escalating epidemic trends throughout the tropical and subtropical regions of the world [9,10]. This is attributed to the expansion of the vectors’ geographic range, global travel, unplanned urbanization and climate change [10]. Since the 1960s, CHIKV has repeatedly been isolated in Africa, Asia and Latin America. In Africa, chikungunya outbreaks have been reported in 25 countries [11]. In Tanzania, chikungunya has been reported in southern, southwestern, northern and central regions of the country [2,12–15].

Tanzania is experiencing a change in etiologies of febrile illnesses as it is for many other African countries [16]. While the numbers of malarial febrile illnesses are decreasing in sub-Saharan Africa [16], the numbers of non-malarial febrile cases are increasing [17,18]. Arboviruses are considered the likely cause of some non-malarial febrile illnesses and are gaining more prominence as observed through increased mosquito-borne viral disease epidemics [12,15,19,20]. Recent studies in Tanzania have revealed that patients with acute dengue and chikungunya infections are often misdiagnosed and treated with antimalarials or antibiotics, mainly due to lack of differential diagnosis [21]. Patients with non-malarial febrile illnesses such as chikungunya may present with clinical signs similar to those of other febrile illnesses, thus laboratory confirmation is essential but often lacking in low-income countries [7,22].

The paucity of information on the epidemiology of chikungunya in Tanzania has limited its prevention and control. Thus, the objective of this study was to determine the seroprevalence and the burden of chikungunya among febrile patients seeking medical care from healthcare facilities in selected districts of Tanzania.

## Materials and methods

### Study sites

**Figure 1.**
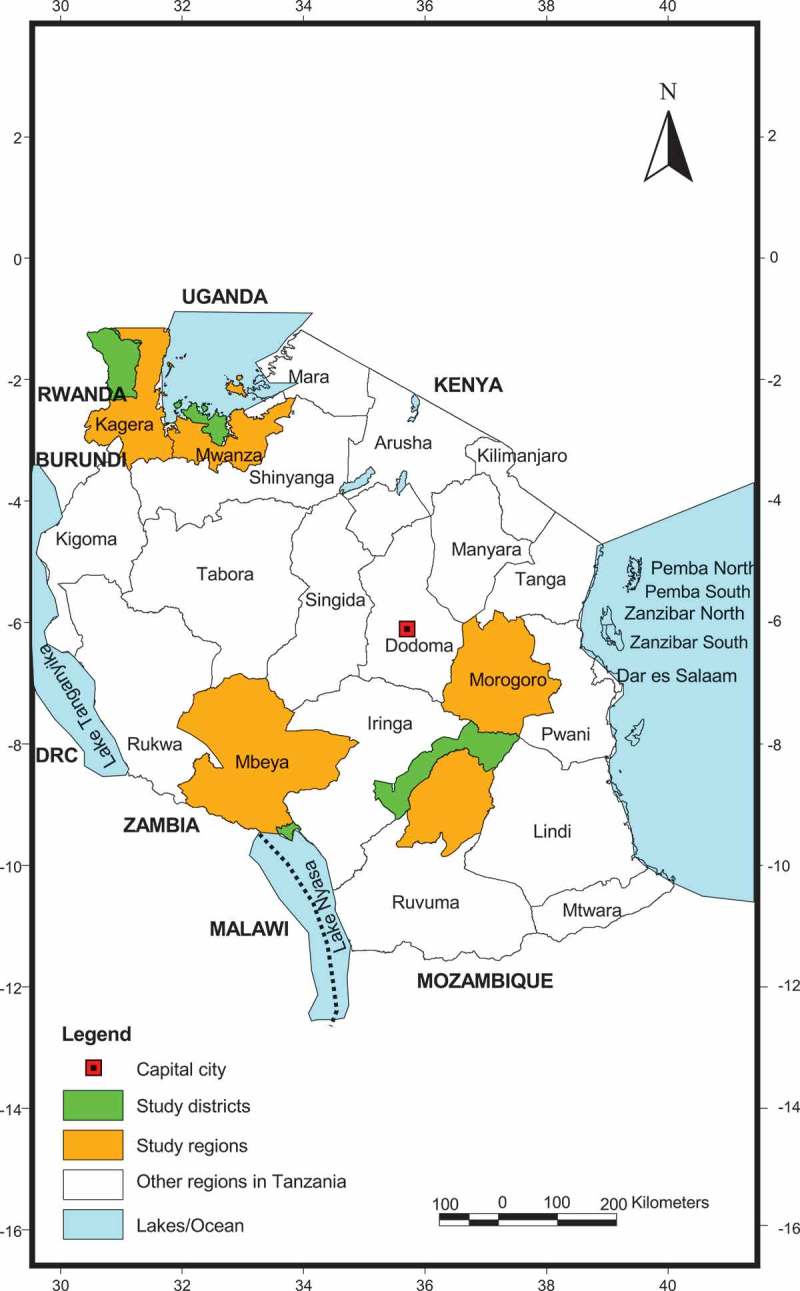
Map of Tanzania showing study locations which included four districts (highlighted in green) located within four regions (highlighted in yellow).

The study was carried out in four regions of Tanzania (Kagera, Mwanza, Morogoro and Mbeya). These regions were selected based on variation in their altitudes, climate and, the prevalence of fever and malaria [21,23]. In each region, one district was randomly selected for the study (Figure 1).

Karagwe district is characterized by mountain ranges with swampy valley bottoms and wetlands. The altitude ranges from 1,500–1,800 m above sea level. The annual average temperature is 26°C while rainfall distribution is bi-modal with peak rains between September and December and March and May. Most parts of Karagwe district receive average rainfall between 800 mm and 1,000 mm annually. The vegetation ranges from a thick equatorial type of forest in the northern part to predominantly savannah bushes in the southern part. Karagwe vegetation landscape is dominated by grasslands and banana plantations. The major economic activities are subsistence agriculture, fishing and livestock keeping.

Sengerema district in Mwanza region is located in south-western part of the Lake Victoria basin. The district lies at 1,100–1,450 m above sea level, and the inland area is covered by seasonal rivers and streams flowing into Lake Victoria. The area is characterized by a tropical climate, with an average annual temperature of 26.5°C, and experiences long rainy seasons between January and May. Annual average rainfall is 1,065 mm. The majority of inhabitants are involved in rural subsistence agriculture, fishing and livestock keeping.

Kilombero district in Morogoro Region is located at an elevation of 700–1,700m above sea level within the Kilombero river valley which is seasonally flooded up to 52 km wide at high waters. The district is characterized by a short rainy season from October to December and a long one from February to June. Annual rainfall ranges from 1,200 to 1,800 mm and the annual mean temperature is about 26°C. The Kilombero valley has a diverse ecology and demography with villages extending to the margins of flood plain where rice cultivation is the principal economic activity.

Kyela district lies on the flood plains (490–520 m) of Lake Nyasa and thus receives heavy rains of about 3,000 mm per year. Kyela has a hot and humid climate with a mean daily temperature of 23 °C. The rainy season is between November and June, with the heaviest rains falling in April-May. The natural vegetation is of tropical savanna forest and grasslands with lagoon vegetation on the swamps and river mouths to the Lake Nyasa. Crop agriculture (cocoa, coffee, tea, cardamom, rice, banana, bean, maize) is the main economic activity of the people in Kyela district.

### Study design and participants

This facility-based cross-sectional study was carried out during and immediately after the rainy season in May-June 2015. Based on a large turnout of outpatients, we deliberately selected Nyakahanga District Designated Hospital (Karagwe), Mang’ula Health Centre (Kilombero), Sengerema District Hospital (Sengerema) and Kyela District Hospital (Kyela). Study participants were patients meeting the study criteria who presented to the health facility on all days and at any hour of the day during the study period. Eligibility criteria included the presence of acute febrile syndrome (axillary temperature ≥38 °C, age > 1 year and history of fever in the past >2 days. The exclusion criteria were participants with chronic diseases or severe illnesses, which required immediate or inpatient treatment as was determined by clinicians. For equal statistical representation, enrollment was capped at 182 eligible febrile patients at each study site.

### Collection of socio-demographic data

A structured questionnaire was administered to collect socio-demographic data of the study subjects. Upon recruitment of eligible subjects, a clinician documented sex and age of the participant and asked questions on the current residence, duration of stay in the residence, travel history, occupation, contact and mode of contact with wild animals and/or mosquito bite frequencies.

### Clinical examination and sample collection

The clinical history, including the history of fever, and physical examination were performed by a trained clinician of the respective health facility. The clinician recorded symptoms associated with febrile illness and findings obtained after performing a standard clinical examination of the participant. The clinical diagnosis and patient management were done according to the national guidelines and were recorded on a standard assessment form. Five millilitres of venous blood was collected aseptically into tubes with anticoagulant-ethylene-diamine-tetra-acetic-acid (EDTA) and plain tubes from all study subjects. All samples were temporarily stored at −20 °C at each of the health facilities before being transported to the laboratory at the Sokoine University of Agriculture, where they were kept at −80°C until analyzed.

### Serological assays

Enzyme-linked immunosorbent assay (ELISA) was performed for the detection of IgG and IgM antibodies against CHIKV (Euroimmun AG, Luebeck, Germany), as per manufacturer’s instructions.The quality of the antigen used in this Euroimmune AG-ELISA ensures a high specificity of the ELISA. In comparison with anti-chikungunya virus indirect immunofluorescence test, the diagnostic specificity and sensitivity of IgG test were 98.6% and 95.4% and, IgM test were 98.9% and 98.1% respectively. The borderline test results, a secure evaluation was not possible with no alternative test method and follow up samples. Thus, equivocal results (≥16RU/ml to ˂22 RU/ml) obtained with the serological assays were grouped with the negative results (˂16RU/ml). The reliability of the ELISA was improved by duplicate determinations for each sample. Both calibrators (C1, C2 and C3), positive, and negative controls served as internal controls for the reliability of the test procedure. They were assayed with each test run.

### Data analysis

Sample size to estimate the seroprevalence of chikungunya in febrile patients was computed based on the following assumptions: (i) expected seroprevalence of 7.9% was used as the basis for comparison, the seroprevalence reported in recent studies [14,15,19,23] conducted in Tanzania and; (ii) precision rate of 4%. Data collected from the questionnaires were entered and analyzed using Microsoft Office-Excel 2007 (Microsoft, California, USA) and Epi Info version 7.0.8.0 (CDC, Atlanta, USA). Age, sex, and locality distribution of the seropositive and seronegative subjects were compared using Chi-square test. All tests were statistically significant at P ≤ 0.05.

## Results

### Demographic and clinical characteristics of the study participants

A total of 1,310 outpatients were involved in this study. Of these, 728 (55.6%) met the study criteria and were recruited for the study. Of the 728 febrile patients, 266 (36.5%) were males and 462 (63.5%) were females, with the mean age of 28.8 years. Most of the febrile subjects (n = 184) were in the age group 20–29 years, followed by 169 in 10–19 years; 116 in 30–39 years; 106 in ≥50 years; 87 in 1–9 years and 66 in 40–49 years, respectively.

The most common symptoms reported among febrile patients included fever (100.0%), headache (70.0%), joint pains (68.3%), vomiting (61.5%), leg weaknesses (59.5%), laboured breathing (58.0%) and abdominal pains (41.8%). The least observed signs were neck stiffness (33.8%) and skin rashes (26.0%) (Table 1). Joint pains were prolonged with time length ranged from 3 to 90 days, with a mean of 7 days, predominantly affecting ankle joints, elbows and knees. Among all the patients, 75.0% reported the absence from work or school because of fever and joint pains.

**Table 1.** Clinical features of febrile patients in Karagwe, Sengerema, Kilombero and Kyela districts of Tanzania.

	Number of subjects (%)		
Clinical symptoms/signs	Yes	No	Unknown
Fever	728 (100.00)	0 (0.0)	0 (0.0)
Headache	510 (70.0)	200 (27.5)	18 (2.5)
Nausea	402 (55.3)	290 (39.8)	36 (5)
Vomiting	448 (61.5)	280 (38.5)	0 (0.0)
Joint ache	497 (68.3)	231 (31.8)	0 (0.0)
Stiff neck	246 (33.8)	426 (58.5)	56 (7.8)
Seizures	264 (36.3)	457 (62.8)	7 (1.0)
Leg weaknesses	433 (59.5)	246 (33.8)	49 (6.8)
Skin rash	189 (26.0)	539 (74.0)	0 (0.0)
Abdominal pains	304 (41.8)	424 (58.3)	0 (0.0)
Pallor	395 (54.3)	333 (45.8)	0 (0.0)
Jaundice	411 (56.5)	317 (43.5)	0 (0.0)
Laboured breathing	422 (58.0)	306 (42.0)	0 (0.0)

### Serology for CHIKV infection

A total of 728 serum specimens were analyzed for anti-CHIKV IgG and anti-CHIKV IgM antibodies. . Of these specimens analyzed, 105 (14%) tested positive for anti-CHIKV IgG antibodies only, whilst 11(1.5%) tested positive for anti-CHIKV IgM antibodies only and 13 (1.8%) samples tested were equivocal (Table 2). There was a higher IgG seropositivity in Karagwe (26.9%) than in Sengerema (12.6%), Kilombero (10.4%) and Kyela (7.7%) (Table 2). The proportion of IgM seropositive cases were very low compared to their counterpart IgG among districts (Table 3).

**Table 2.** Seroprevalence of anti-CHIKV-IgG among febrile patients in Karagwe, Kilombero, Kyela and Sengerema districts.

Districts	No. of patients	No. of IgG positive	Seropositivity (%)	Seronegativity (%)
Karagwe	182	49	49 (26.9)	133 (73.1)
Kilombero	182	19	19 (10.4)	163 (89.6)
Kyela	182	14	14 (7.7)	168 (92.3)
Sengerema	182	23	23 (12.6)	159 (87.4)
Total	**728**	**105**	**105 (14.4)**	**623 (85.6)**

**Table 3.** Comparison between clinical features and demographic characteristics of study participants with acute chikungunya infection in Karagwe, Kilombero, Kyela and Sengerema districts.

Characteristics	Acute CHIKV infection Overall seropositivity: 11/728 (1.5%)		No acute CHIKV infection Overall seronegativity: 717/728 (98.5%)	
Total	n = 11	%	n/N	%
**Demographics**				
Gender				
Male	2	(18.2)	264/266	99.2
Female	9	(81.8)	453/462	98.1
Districts				
Karagwe	5	(45.5)	177/182	97.3
Kilombero	2	(18.2)	180/182	98.9
Kyela	3	(27.3)	179/182	98.4
Sengerema	1	(9.1)	181/182	99.5
**Clinical signs and symptoms**				
Headache	3	(27.3)	507/510	99.4
Nausea	0	(0.0)	402/402	100
Vomiting	1	(9.1)	447/448	99.8
Joint ache	7	(63.6)	490/497	98.6
Stiff neck	0	(0.0)	246/246	100
Seizures	0	(0.0)	264/264	100
Leg weaknesses	2	(18.2)	431/433	99.5
Skin rash	6	(54.5)	183/189	96.8
Abdominal pains	1	(9.1)	303/304	99.7
Pallor	0	(0.0)	395/395	100
Jaundice	0	(0.0)	411/411	100
Laboured breathing	3	(27.3)	419/422	99.3

The highest seropositivity (35.9%) was found among the 20–29 years age group accounting 71 seropositive chikungunya febrile patients. Participants within 30–39 years age group were more likely to be seropositive than those under 20 years (Table 4). The proportion of participants who were IgG seropositive was higher than the proportion of participants who were IgM seropositive for all age groups. Among participants of the 20–29 years group, 66 out of 184 had anti-CHIKV IgG antibodies detected, which was significantly higher than the proportion of participants of 30–39 years old with IgG antibodies (Table 4). However, there was no statistical difference in the presence of anti-CHIKV IgM antibodies among the febrile participants under 19 years (P ≤ 0.05). The most of chikungunya seropositive participants reported to have been bitten by mosquitoes during the day and hospitalized or confined to bed at home for an average of 7 days (range of 3 to 60 days).

**Table 4.** The proportion of seroprevalence of chikungunya virus infection by age group.

Age group (years)	No. of participants	No. (%) of IgG positive	No. (%) of IgM positive	*P* value
1–9	87	3 (3.5%)	1 (1.2%)	0.3
10–19	169	11 (6.5%)	4 (2.4%)	0.06
20–29	184	66 (35.9%)	5 (2.7%)	<0.001
30–39	116	19 (16.4%)	1 (0.9%)	<0.001
40–49	66	2 (3.0%)	0 (0.0%)	-
≥50	106	4 (3.8%)	0 (0.0%)	-

## Discussion

Diagnosis of mosquito-borne viral diseases in resource-poor settings is challenging. In malaria endemic areas most febrile illnesses have been and are treated as malaria cases due to lack of diagnostic capacity to properly rule-out malaria as a cause of fever and as well as identify alternative fever-causing pathogens [24]. As for malaria, diagnosis of chikungunya based on clinical presentation is difficult [24,25]. Furthermore, similar presentations of signs and symptoms in the initial stage of illness of chikungunya have been a challenge to clinically distinguish it from other mosquito-borne viral diseases [26]. Although the vast majority of clinical chikungunya cases are self-limiting, diagnosis of patients with the infection is important for prompt appropriate case management.

The variation in the seroprevalence observed between the districts is most likely to be associated with the ecological and topographical characteristics that support mosquito breeding. Interestingly, higher seroprevalence rates were among patients in Sengerema and Karagwe districts which are on the plateau at over 1,100 m above sea level. In a recent study in Nepal, vectors of chikungunya, *Aedes aegypti* and *Ae. albopictus* were observed from low (80 m) to high (2,100 m) altitude [27]. It has been described that the favourable habitats that are available for vector mosquitoes differ with climatic variables (rainfall, temperature and relative humidity) and vegetation [27].

In addition, while Karagwe is characterized by the banana plantations, which are most likely to contribute highly in providing breeding sites for *Aedes* mosquitoes, the other three districts are characterized by rice farming that provides large bodies of water, likely to be suitable for mosquito breeding. Nonetheless, there is scarce information on mosquito vectors and their CHIKV transmission dynamics in all the districts, except for Kyela [28,29]. In a recent study in Kyela, CHIKV was detected in *Ae. aegypti* mosquitoes that were collected from areas where rice farming was common [28]. *Ae. aegypti* has been reported as a common outdoor breeding mosquito in Kilombero district [30] with clay pots, buckets, tins, drums used for storing water and tree holes as the common breeding sites. Studies on entomological dynamics are recommended to establish chikungunya transmission indices in the areas.

Based on our findings there are indications that some patients had previous exposure to CHIKV. This finding is important to understand the signals of chikungunya in different parts of Tanzania. Previous studies have also indicated the occurrence of presumptive and prior exposure to CHIKV infections in the country [14,15,22,31]. Our results showed that anti-CHIKV IgM antibodies were found among a very few patients within 7 days after the onset of fever hence suggesting possibility of acute infection (presumptive acute CHIKV infections). Most often, in acute CHIKV infections IgM antibodies appear first and can be detected during the first week of the disease particularly from day 5 while IgG antibodies appear shortly afterwards [31–34]. The detection of IgM antibodies in patients who had a history of fever for less than 5 days, possibly indicate a recent CHIKV exposure. The IgM response tends to decline within approximately 3 to 4 months, although low levels can persist for 1 year or more whilst IgG antibodies are much more long-lasting [35]. The presence of CHIKV specific IgM antibodies, with or without CHIKV specific IgG antibodies, signifies current or very recent infection; and the presence of CHIKV-specific IgG, but not IgM antibodies, signifies past infection [35,36]. The latter pattern often, but not always, indicates immunity to subsequent infection. Similarly, in some patients, the IgM antibody response is transient or low level, leading to failure to detect virus-specific IgM antibodies, decreasing the specificity of the assay as an indicator of very recent infection [36]. Thus, a negative serological result does not exclude an infection. Since this study was cross-sectional, a cautious interpretation of this finding is necessary, particularly in the absence of comparison between acute and convalescent serum from the same patient.

The overall seroprevalence of chikungunya among febrile participants is the highest to be reported in Tanzania. Previous studies in central and northern Tanzania reported relatively lower prevalence rates [14,19]. Also our results revealed a high IgG seropositivity (14%) which is three times higher than previously reported in central Tanzania [14]. Since there is no routine diagnosis for chikungunya at the health facilities in Tanzania, it is likely that such infections go unnoticed or when causing fever end up being treated as other common infections such as malaria or bacterial infections, leading to underreporting and underestimation of the burden [14].

Moreover, we observed that the number of chikungunya IgG seropositives were higher than the number of IgM seropositives. While the IgM positivity did not differ among children and adults, IgG positivity was significantly higher in adults than in children. The presence of IgM indicates active circulation of chikungunya in the study sites, with adults having a prior exposure which also points to a possible endemicity with varying cumulative increases in IgG seropositivity with age.

Clinical features associated with CHIKV infection such as fever, joint pains, and myalgia are non-specific and could be mistakenly identified with a variety of other diseases including dengue, malaria, Rift Valley fever, and influenza, which are endemic in Tanzania [13,20,33]. However, pronounced persistent severe joint pains that affect wrists, elbows, fingers, and knees in some patients should raise the suspicion of alphavirus infections, especially chikungunya. Therefore, we recommend the establishment of a better diagnosis of fever to avoid over-treatment of other diseases and drug resistance.

## Conclusions

The present study was conducted to investigate the extent of seropositivity to chikungunya virus amongst febrile patients seeking healthcare in Tanzania. The findings indicate circulating CHIKV in Karagwe, Kilombero, Kyela and Sengerema districts. High seroprevalence of CHIKV was found affecting the economically active age group. Further studies are required to estimate the entomological transmission indices and its prevention and control strategies.

## Declarations

***Ethics considerations***

The study was approved by the Tanzania Medical Research Coordinating Committee of the National Institute for Medical Research (Approval number NIMR/HQ/R.8a/Vol.1X/1946). Consent was sought from all potential candidates and those meeting all inclusion criteria and willing to participate were recruited after signing a written informed consent. Consent was obtained from the parents or guardians for those children under 18 years of age.

***Consent for publication***

Not applicable.

***Availability of data and materials***

The dataset analyzed during the current study are available from the corresponding author on reasonable request.
